# Post traumatic stress disorder among patients who survived COVID 19

**DOI:** 10.1192/j.eurpsy.2023.372

**Published:** 2023-07-19

**Authors:** I. Trabelsi, R. Youssef, O. hwichi, A. Loghmari, H. Yaacoubi, K. Bouassida, L. Boukadida, R. Jaballah, H. Ben Salah, A. Zorgati, R. Boukef

**Affiliations:** ^1^Emergency Department; ^2^Urology, Sahloul teachin Hospital Sousse, Sousse, Tunisia

## Abstract

**Introduction:**

Research on past coronavirus outbreaks, including Severe Acute Respiratory Syndrome (SARS) and Middle East Respiratory Syndrome (MERS), indicates a high likelihood of symptoms and psychiatric disorders in survivors, including symptoms of stress. post-traumatic stress disorder (PTSD) and post-traumatic stress disorder (PTSD)

**Objectives:**

This study therefore set out to highlight the impact of COVID-19 infection on mental health by screening them for posttraumatic stress

**Methods:**

It is a retrospective study that includes patients who were tested positive with COVID 19 (RT PCR using a nasopharyngeal test ) and who have seeked medical care at emergencies during a month . The post traumatic stress score disorder: PCL-5 was calculated on day 30 and day 90. The evolution of the patients health state (Recovery or deterioration) was marked. All data were analyzed by SPSS.

**Results:**

we included during the study period 200 patients complying with the inclusion criteria. Post traumatic stress was diagnosed in 146 patients (73% of patients). Post-traumatic stress was diagnosed in rather elderly patients; the average age was 51.8 years with a female predominance (57.5%). 63.6% of patients with PTSD had a cough; 35.7% had dyspnea; 49.7% were febrile, 43.9% had arthromyalgia, 15% had anosmia.The disappearance of the signs was after 8.3 days on average, it lasted longer (14.6 days) in the patient who developed PTSD.In this series, 47.3% of patients diagnosed with PTS infected their relatives

**Conclusions:**

This study contributes to a better understanding of the factors that determine the impact of traumatic events such as a pandemic on people’s mental health.Post traumatic stress disorder is so common among COVID 19 patients and it has a huge influence on the evolution of their health state . This is why all health workers have to fight against COVID and its effects on both physical and mental health . Highlighting the fact that a psychological assistance is highly recommended in the management ofCOVID19 patients in order to improve their prognosis

**Disclosure of Interest:**

None Declared

